# Obese donor mice splenocytes aggravated the pathogenesis of acute graft-versus-host disease via regulating differentiation of Tregs and CD4^+^ T cell induced-type I inflammation

**DOI:** 10.18632/oncotarget.20425

**Published:** 2017-08-24

**Authors:** Zengyao Li, Jian Gu, Qin Zhu, Jing Liu, Hao Lu, Yunjie Lu, Xuehao Wang

**Affiliations:** ^1^ Liver Transplantation Center, First Affiliated Hospital, Nanjing Medical University, Nanjing 210029, China; ^2^ Department of Radiotherapy, First Affiliated Hospital, Nanjing Medical University, Nanjing 210029, China

**Keywords:** obesity, aGVHD, transplantation

## Abstract

Acute graft-versus-host disease (aGVHD) remains one of the most severe complications in organ and bone marrow transplantation, leading to much morbidity and mortality. Obesity has been associated with increased risk of development of various inflammatory diseases. Here, we investigated the *in vitro* and *in vivo* effects of obese donor splenocytes on the development of acute graft-versus-host disease (aGVHD). In this study, mixed lymphocyte reactions (MLR) *in vitro* showed that obese donor mouse CD4^+^ T cell promoted the production of interleukin-2 (IL-2), interferon (IFN)-γ and tumor necrosis factor (TNF)-α. Meanwhile, the inducible Tregs population decreased greatly in obese donor mouse CD4^+^ T cells’ induction group, compared with normal group. Then in the murine aGVHD model, we found that obese donor splenocytes dramatically increased the severity of aGVHD through down-regulating immune tolerance while enhancing systemic and local immunity. Moreover, we showed that aGVHD induced by obese donors resulted in massive expansion of donor CD3^+^ T cells, release of Th1-related cytokines, interleukin-17 (IL-17) and chemokines, significant increase of Th17 cells and inhibition of CD4^+^CD25^+^Foxp3^+^ regulatory T cells (Tregs) and impaired suppressive ability of donor Tregs. Expression of sphingosine-1-phosphate receptor 1 (S1PR1), phosphorylated Akt, mammalian target of rapamycin (mTOR) and Raptor increased, while the phosphorylation level of SMAD3 was decreased in the skin, intestine, lung and liver from obese donor splenocytes-treated aGVHD mice. Furthermore, at mRNA and protein levels, we defined several molecules that may account for the enhanced ability of obese donor splenocytes to migrate into target organs, such as IL-2, IL-17, IFN-γ, TNF-α, CXCR3, CXCL9, CXCL10, CXCL11 and CCL3. Therefore, these results imply that obese donor cells may be related to the risk of aGVHD and helping obese donor individuals lose weight represent a compulsory clinical strategy before implementing transplantation to control aGVHD of recipients.

## INTRODUCTION

Acute graft-versus-host disease (aGVHD) is a major complication following allogeneic hematopoietic cell transplantation (allo-HCT) and tissue transplantation from genetically different person, limiting the success of this therapy [1]. As a complex immunopathology, aGVHD hinges on recognition of host alloantigens by donor T cells and results in the expansion of alloreactive T cells and various inflammatory cytokines [2–4]. Recently, several pro-inflammatory cytokines secreted following the conditioning regimen, such as interleukin-2 (IL-2), tumor necrosis factor (TNF)-α, IL-12, interferon (IFN)-γ and CXCL9, have been connected to the initiation and pathophysiology of aGVHD [5–10]. Several studies also observed that aGVHD was induced by type I inflammation based on the predominant increased production of the cytokines, including IFN-γ and TNF-α [11, 12]. Moreover, the expansion of CD4^+^CD25^+^Foxp3^+^ regulatory T cells (Tregs) plays an important role in suppressing aGVHD [13].

Obesity, defined as the excess accumulation of intra-abdominal body fat and an epidemic of the twenty-first century, continues to rise throughout the world, even in the countries where poverty and malnutrition are major problems. The World Health Organization estimates that globally there are more than 1.9 billion overweight adults (body mass index (BMI) > 27 kg/m2). Of them, 600 million people are obese with BMI more than 30 kg/m2 (WHO obesity and overweight fact sheet, updated in June 2016: http://www.who.int/mediacentre/factsheets/fs311/en/) [14]. Obesity has been linked with increased incidences of infectious diseases, including periodontal infections, bacterial pneumonia and surgical site infections [15]. Recently, obesity was reported to be associated with low-grade systemic inflammation and was identified as a possible risk factor for autoimmune diseases [16–18]. Similarly, a high frequency and expansion of peripheral and in situ conventional CD4^+^ T cells could be observed in overweight individuals, secreting high amount of pro-inflammatory cytokines (IFN-γ, TNF-α) [19]. Moreover, the function of Treg cells has also been reduced remarkably in obese patients [20]. Obesity for recipients is considered a risk factor for aGVHD and increased relapse in HSCT [21–23]. However, since obesity is a global epidemic, whether obesity for donor can affect the development of aGVHD has not been tested to date.

In this study, we investigated the effect and mechanism of obese donor splenocytes on the development of acute graft-versus-host disease (aGVHD). *In vitro* and *in vivo*, we examined whether obese donor splenocytes affected aGVHD severity, donor cells expansion, pro-inflammatory cytokine and chemokine expression, and Tregs differentiation. Western blot analysis was established to determine the involvement of Akt/mTOR pathways. We observed that obese donor splenocytes deteriorated the survival of aGVHD while T cell expansion and type I inflammatory cytokine expression were promoted and Tregs differentiation was inhibited. Obese donor CD4^+^ T cell *in vitro* promoted the production of pro-inflammatory cytokine and inhibited Tregs population. CFSE labeling assay *in vitro* showed that proliferative capacity of T cells was increased. Mechanism analysis proved that obese donor splenocytes induced the higher expression of p-Akt and p-mTOR. Our results strongly suggest that controlling obese donor individuals’ weight could be effective in preventing the complications such as aGVHD.

## RESULTS

### Body weight and metabolic parameters in C57BL/6 mice

**Figure 1:**
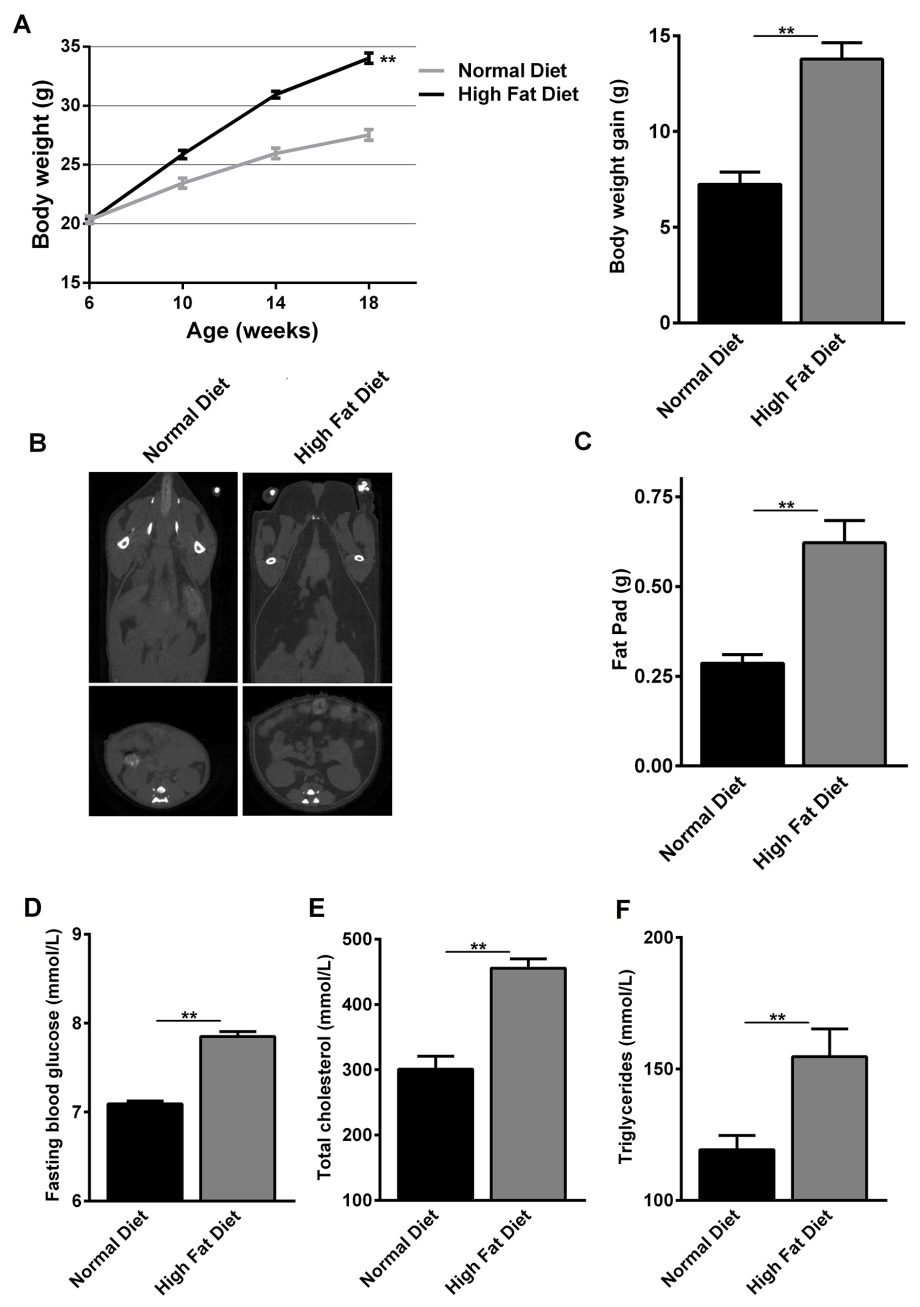
Changes in body weight and metabolic parameters of normal diet- and high fat diet-fed C57BL/6 mice At the beginning of the experiment, 6 weeks old C57BL/6 mice had similar body weights. Then C57BL/6 mice fed normal diet and high fat diet for 12 weeks. **(A)** Body weights at 6, 10, 14 and 18 weeks of age and body weight gain at 18 weeks of age are shown as mean ± SEM of 10 animals per group, ^*^P<0.05 for C57BL/6 mice feeding normal diet versus high fat diet. **(B)** CT images showing fat distribution are representative of three separate analyses performed on mice that had median body mass of their litters. **(C)** Fat-pad mass at 18 weeks of age are shown as mean ± SEM of 10 animals per group, ^*^P<0.05 for C57BL/6 mice feeding normal diet versus high fat diet. **(D-F)** Fasting blood glucose, total cholesterol and serum triglycerides at 18 weeks of age are shown as mean ± SEM of 10 animals per group, ^*^P<0.05 for C57BL/6 mice feeding normal diet versus high fat diet.

To examine whether obese donors can affect the development of aGVHD in a mouse allo-HCT model, we exploited B6 mice to develop diet-induced obesity. At the beginning of the experiment, 6 weeks old C57BL/6 mice had similar body weights. At 18 weeks of age, C57BL/6 mice fed normal diet for 12 weeks, had significantly lower body weight (*p<0.01*), body weight gain (*p<0.001*), fasting blood glucose (*p<0.001*), serum triglycerides (*p<0.001*) and total cholesterol (*p<0.001*) in comparison with C57BL/6 mice fed HFD for 12 weeks (Figure 1A, 1D–1F). Computed tomography (CT) revealed increased fat mass throughout the body of the C57BL/6 mice fed HFD, with a particular increase in visceral fat (Figure 1B). C57BL/6 mice fed HFD had fat pads that were about twice as large as those fed normal diet at 18 weeks of age (Figure 1C). Analysis of these C57BL/6 mice showed that, at 18 weeks of age, C57BL/6 mice fed HFD had body masses that were 20% greater than ones fed normal diet, which is an internationally recognized obese criterion [24]. Hence, these data proved that HFD feeding-induced obesity had been established successfully in C57BL/6 mice.

### Obesity modulates alloreactive T cell responses and CD3^+^ T cell proliferation *in vitro*

**Figure 2:**
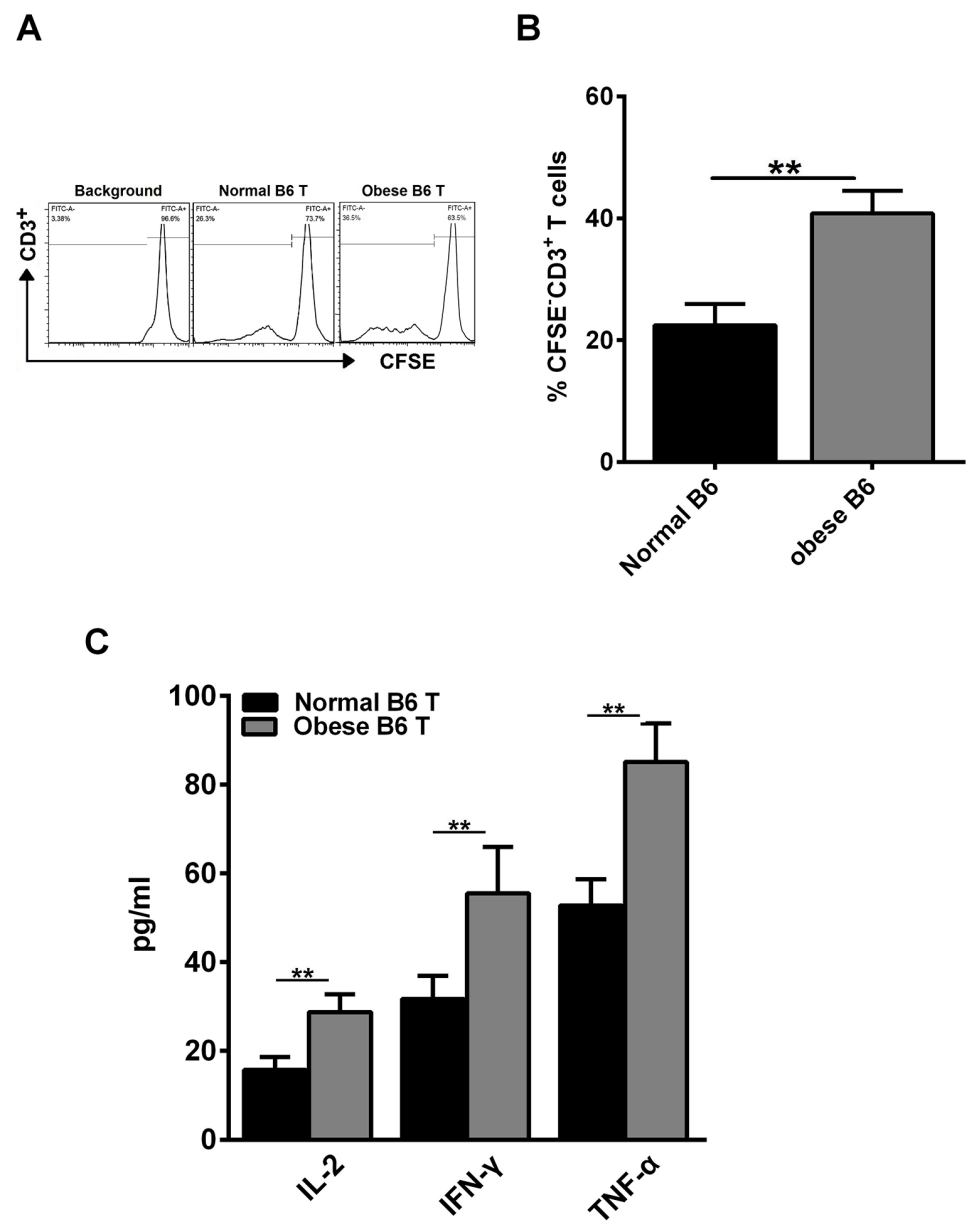
Obesity enhances alloreactive T cell response and CD3^+^ T cell proliferation *in vitro* CD3^+^ T cells from the spleens of normal C57BL/6 and obese C57BL/6 mice were labeled with CFSE and co-cultured with irradiated non-T cells in the presence of anti-CD3 Ab (0.5 μg/ml) for 3 days. Cells were then harvested and analyzed by flow cytometry. **(A)** Representative histogram plot of CD3^+^ T cell proliferation. **(B)** Percentages of CFSE^-^ cells of culture are reported as means ± SEM. Splenocytes derived from BALB/c mice were used as stimulator cells and those from normal C57BL/6 or obese C57BL/6 mice were used as responder cells in MLR assays. Cell culture supernatant for each group was collected at day 3 after culture, the concentrations of IL-2, IFN-γ and TNF-α **(C)** were measured using the sandwich ELISA, ^**^P<0.01. Data are combined from three independent experiments, ^*^P<0.05, ^**^ *P<0.01*.

As aGVHD is induced by the activation of host-reactive donor T cells, persistence of alloreactive T cells is required for the development of aGVHD [25]. Therefore, we investigated whether obesity could enhance alloreactive T cell response as well as CD3^+^ T cell proliferative capacity. Our study showed that the proliferative capacity of CD3^+^ T cells was increased in obese C57BL/6 mice group, compared with normal group (Figure 2A–2B). Meanwhile, concentration of IL-2, IFN-γ and TNF-α in cell culture supernatants was measured using a sandwich ELISA. We found that a significant increase of IL-2, IFN-γ and TNF-α was detected in obese C57BL/6 mice group, compared with normal group (Figure 2C). These data proved that obesity enhanced CD4^+^ T cell induced-type I inflammation *in vitro*.

### Obese donor increases aGVHD–induced lethality and target organ injury of recipients

**Figure 3:**
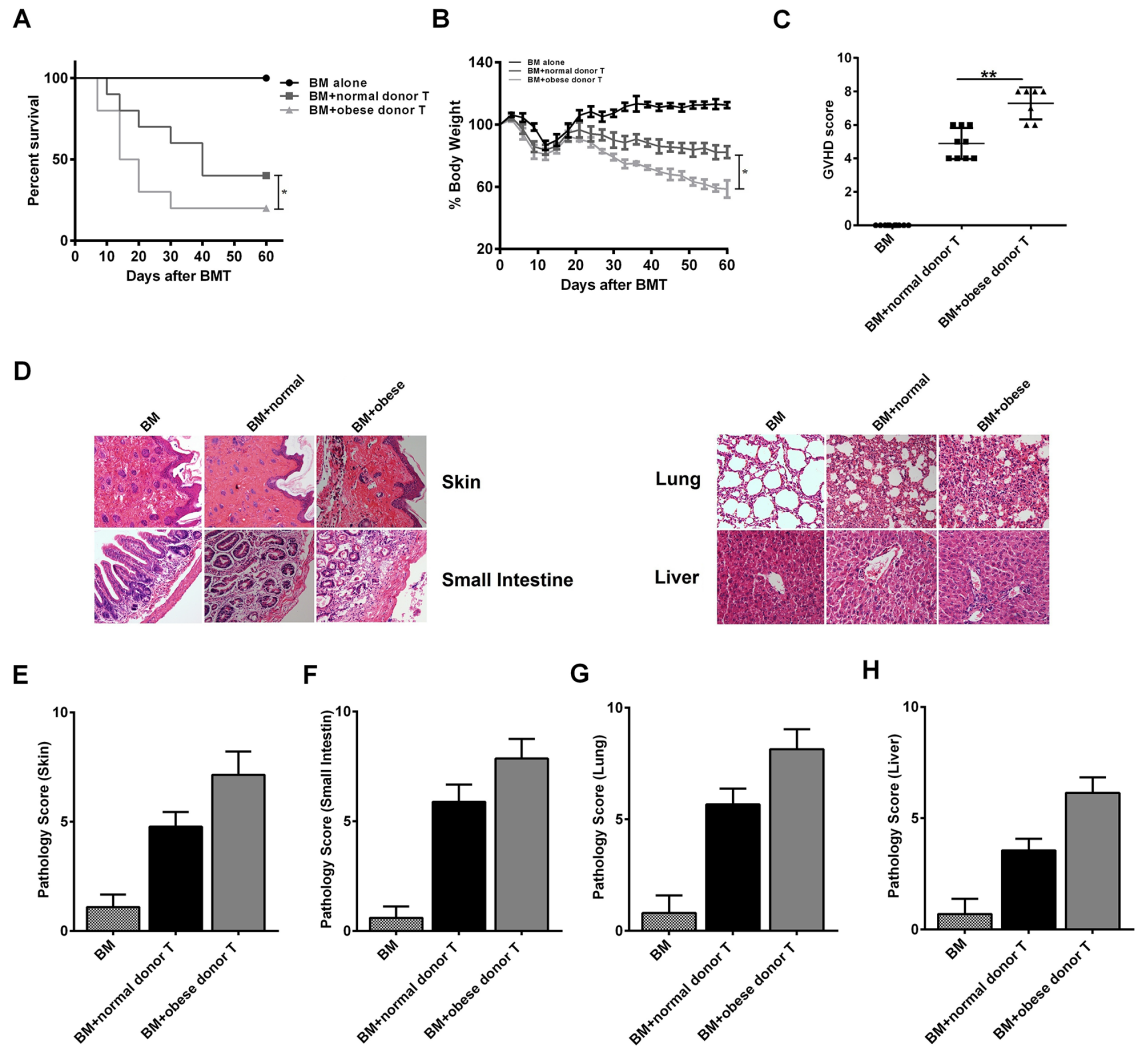
Obese donor splenocytes increases aGVHD–induced lethality and target organs injury Lethally irradiated BALB/c recipients were transplanted with BM-TCD, with or without splenocytes from normal or obese donor mice respectively. The condition for each group was examined every three days. Survival data **(A)** and weight change **(B)** are shown (log-rank test, ^*^P<0.05 for BM-TCD plus WT splenocytes versus BM-TCD plus CHI3L1-KO splenocytes). **(C)** GVHD clinical scores were measured as described in Materials and Methods at days after 14 allo-HCT, ^**^P<0.01. **(D)** Representative plots for skin, small intestine, lung and liver of aGVHD mice at day 14 (×100 magnification). **(E-H)** Pathology scores in the skin, small intestine, lung and liver in mice 14 days after allo-HCT. ^**^P<0.01. Data are shown as mean ± SEM from 3 independent experiments. Each group includes 10 mice.

To examine the contribution of obese donor splenocytes in regulating the development of aGVHD, we induced aGVHD by transferring bone marrow cells and splenocytes from normal C57BL/6 (B6, Normal) or obese C57BL/6 (B6, Obese) mice with T cell-depleted (TCD) BM into lethally irradiated allogeneic BALB/c mice. The recipients in obese donor group showed significantly lower long-term survival rates (median survival time 17 versus 40 days, ^**^ *P>0.01*; Figure 3A), more weight loss (Figure 3B), and higher GVHD clinical scores (^**^P<0.01, Figure 3C) two weeks following allo-BMT, compared with the recipients in normal donor group. Furthermore, we assessed the pathological scores for each group two weeks following allo-BMT, which showed that the histological grade was all significantly increased in the skin (Figures 3D and 3E), the small intestine (Figures 3D and 3F), the lung (Figures 3D and 3G) and the liver (Figures 3D and 3H) of mice that received obese donor splenocytes compared to mice that received normal donor splenocytes. As expected, we observed a significant increase of pathological scores in tissues from mice receiving allogeneic obese donor splenocytes as compared with normal ones, reflecting the increased severity of aGVHD induced by obese donor splenocytes. These data proved that obese donor increased aGVHD mortality and severity after allo-HCT.

### Obese donor promotes the expression of inflammatory mediators in recipients systemically and locally

**Figure 4:**
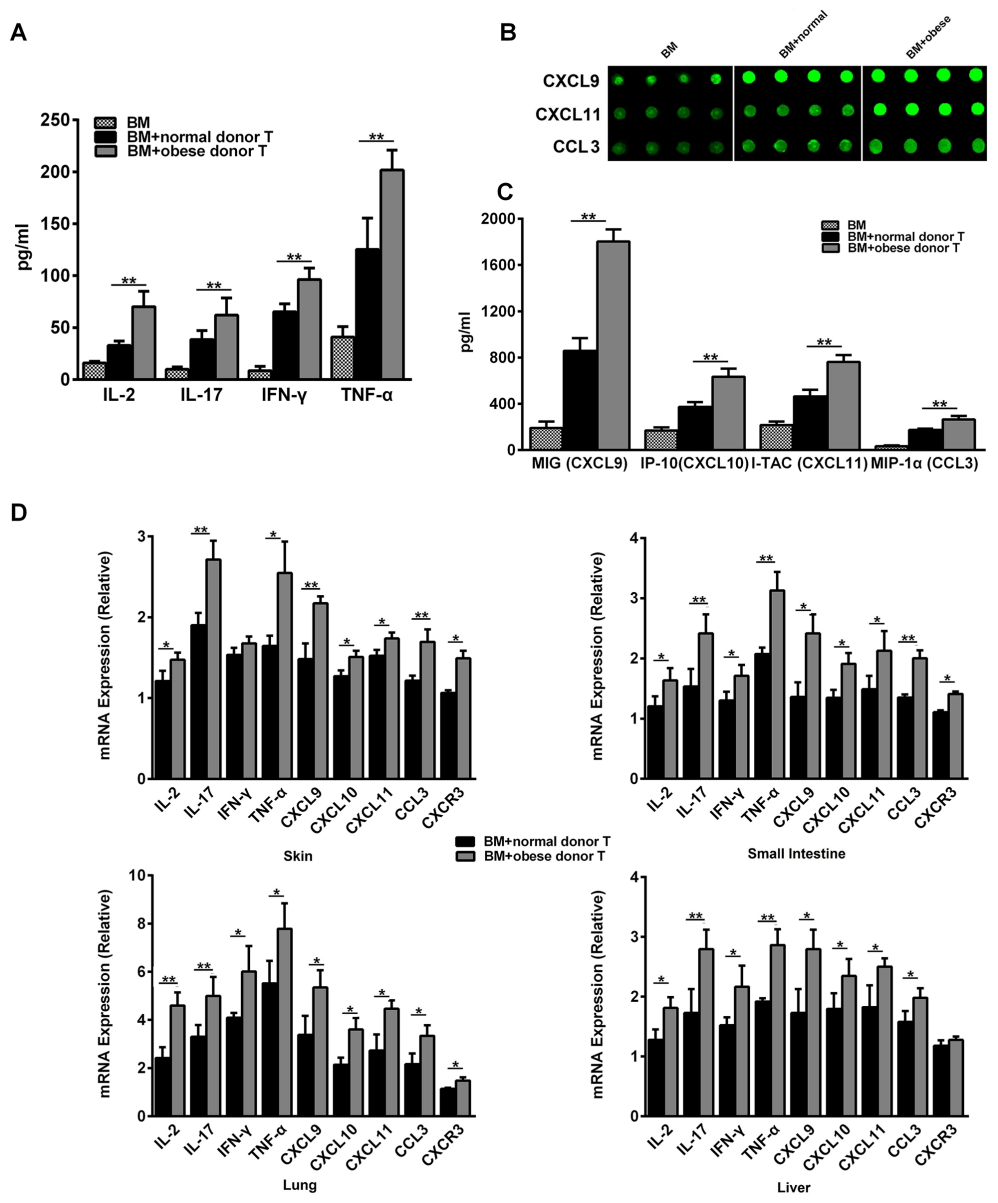
Obese donor enhances the expression of pro-inflammatory mediators in recipients systemically and in aGVHD targeted organs The aGVHD model for each group was built as described above. Blood serum for each group was collected at day 14 after allo-HCT, the concentrations of IL-2, IL-17, IFN-γ and TNF-α **(A)** were measured using the sandwich ELISA, ^**^P<0.01. **(B)** The chemokine profiles for aGVHD were screened using the RayBio Mouse chemokine antibody array and ELISA assay. The signal of CXCL9, CXCL10, CXCL11 and CCL3, were showed. **(C)** Signal counts for chemokines CXCL9, CXCL11 and CCL3 by the chemokine antibody array were presented. Data are presented as the mean ± SEM, ^**^P<0.01. **(D)** Real-time PCR analysis for each indicated genes was performed using RNA isolated from the skin, small intestine, lung and liver collected at day 14 after allo-HCT. Mean ± SEM of gene expression levels is represented. Data are combined from three independent experiments, ^*^P<0.05, ^**^ *P<0.01*.

To explore the underlying mechanism of obese donor splenocytes in regulating severity of aGVHD, we analyzed the systemic and local inflammatory responses following aGVHD. Since Yi et al. observed that cytokine production by donor cells may contribute to the pathogenesis of aGVHD [26], we next tested level of cytokines in the serum of aGVHD recipient 14 days after the model was established. A significant increase of IL-2, IL-17, IFN-γ and TNF-α was detected in obese donor group, compared with normal group (Figure 4A). We also examined the chemokines in the serum using the RayBio mouse chemokine array. Several chemokines were more highly expressed in obese donor group than in normal group, particularly CXCL9 (C-X-C motif chemokine ligand 9), CXCL10 (C-X-C motif chemokine ligand 10), CXCL11 (C-X-C motif chemokine ligand 11) and CCL3 (C-C motif chemokine ligand 3) (Figures 4B and 3C). These results suggested that circulating IL-2, IL-17, IFN-γ, TNF-α, CXCL9, CXCL10, CXCL11 and CCL3, which were the positive regulators for inflammation, were all involved in the obese donor splenocytes mediated aGVHD aggravation.

Recent study suggest that local inflammatory cytokine production might also play an important role in the biology of GVHD [27], therefore, we also examined the local inflammatory response in aGVHD target organs such as the skin, the small intestine, the lung and the liver. As shown in Figure 4D, we found that IL-2, IL-17, IFN-γ, TNF-α as well as the chemokine CXCL9, CXCL10, CXCL11 and CCL3 were greatly up-regulated in the small intestine, the lung and the liver from obese donor groups. While in the skin, the expression of IL-2, IL-17, TNF-α, CXCL9, CXCL10, CXCL11 and CCL3 was also ascended in obese donor groups. Except for the liver, chemokine receptor CXCR3 expression was significantly increased in all other target organs from obese donor groups. These data indicated that obese donor splenocytes directed the expansion of pro-inflammatory mediators in multiple organs after allo-HCT.

### Obese donor leads to an increase of CD4^+^ T cells in the spleen of recipients during aGVHD

**Figure 5:**
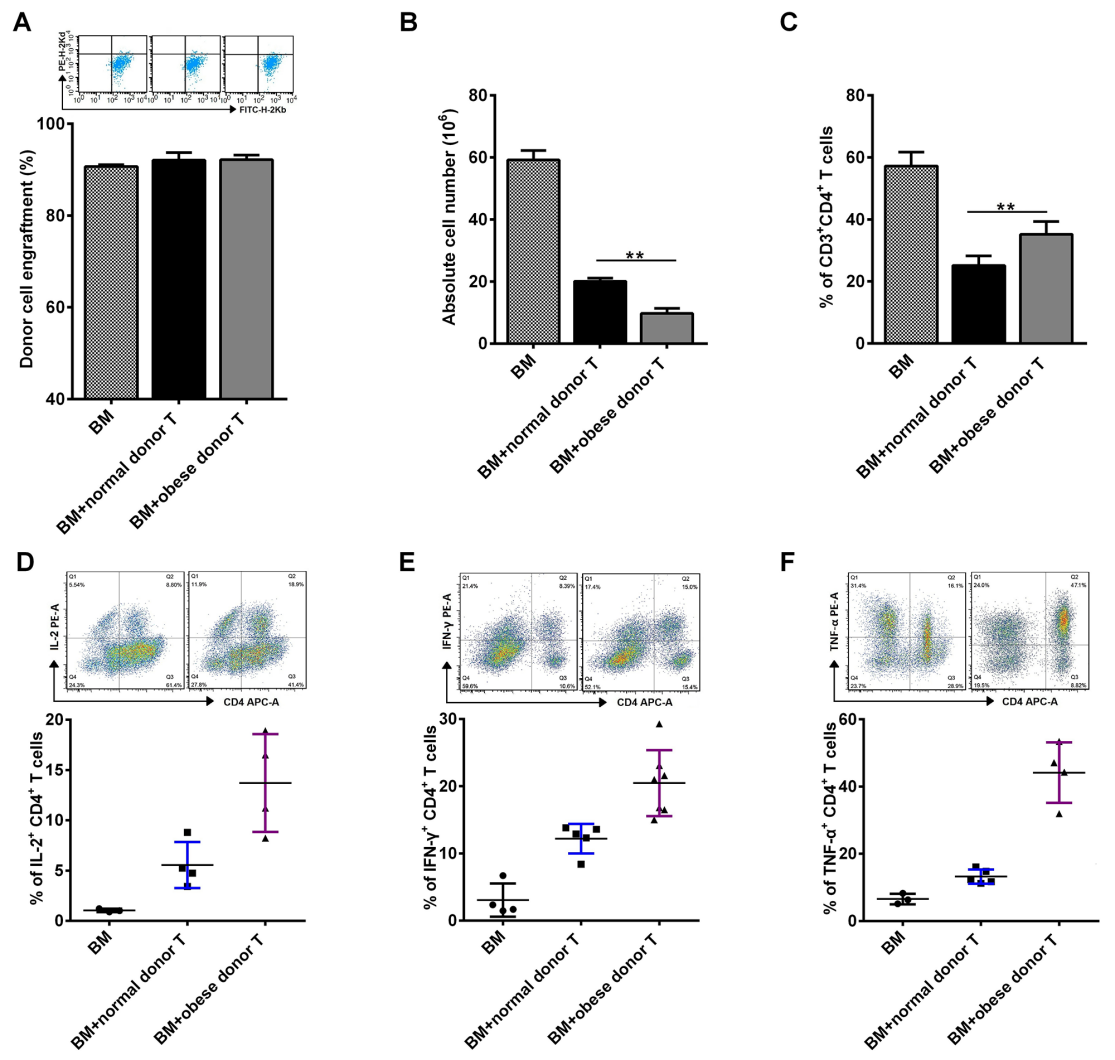
Obese donor splenocytes lead to an increase of CD4^+^ T cells in the spleen of recipients during aGVHD The aGVHD model was built as described above. Splenocytes from recipient mice were harvested at day 14 after allo-HCT. Flow cytometry was used and mean ± SEM of the frequency of engrafted donor cells **(A)**, relative absolute number of splenocytes **(B)** and the proportions of CD3^+^CD4^+^ T cells **(C)** in spleens are shown. **(D-F)** Expression of IL-2, IFN-γ and TNF-α on gated CD3^+^CD4^+^ T cells are shown for a representative splenocytes sample and mean ± SEM of the percentages of IL-2^+^CD4^+^, IFN-γ^+^CD4^+^ and TNF-α^+^CD4^+^ T cells in the spleen is represented. Data are representative of three independent experiments. ^*^ *P<0.05,* ^**^ *P<0.01*.

To determine the *in vivo* function of CD4^+^ T cell subsets during aGVHD, we examined the proportion of CD4^+^ T cells in the spleen of recipient mice by flow cytometry at day 14 after allo-HCT. Firstly, donor and recipient cells can be distinguished by staining H2-Kd and H2-Kb since donor cells are H-2Kb^+^H-2Kd^-^ while recipient cells are H-2Kb^-^H-2Kd^+^. Nearly 90% of donor cells can be observed in the aGVHD group two weeks after cell transfer, which is consistent with the B6→BALB/c aGVHD model reported previously [28]. However, there was no significant difference for the engraftment of donor cells between mice receiving obese or normal splenocytes (Figure 5A). Moreover, it is known that the total number of splenocytes reflects the efficiency of immune cell reconstitution by the donor BM cells in the recipient, which is negatively correlated with the severity of aGVHD [29]. We found that the number of absolute splenocytes was greatly decreased in recipient mice receiving obese donor splenocytes, compared with mice receiving normal donor splenocytes (Figure 5B). In addition, the proportions of CD3^+^CD4^+^ T cells was significantly increased in the spleen of recipient mice receiving obese donor splenocytes, which might indicate that obese donor promoted the expansion of T cells *in vivo* (Figure 5C).

Next, we evaluated the profile of cytokines secreted by alloreactive T cells after allo-HCT. We observed that the secretion of IL-2, IFN-γ and TNF-α was induced in splenocytes from recipients received both normal and obese donor splenocytes (Figure 5D–5F). The proportions of CD4^+^IL-2^+^, CD4^+^IFN-γ^+^ and CD4^+^TNF-α^+^ T cells in spleens were all increased significantly in obese donor group, comparing the normal group, which further implied that the increased secretion of IL-2, IFN-γ and TNF-α may contribute to aGVHD pathophysiology in recipients receiving obese donor splenocytes.

### Obese donor exhibits regulated differentiation of tregs and Th17 as well as tregs functional activity

**Figure 6:**
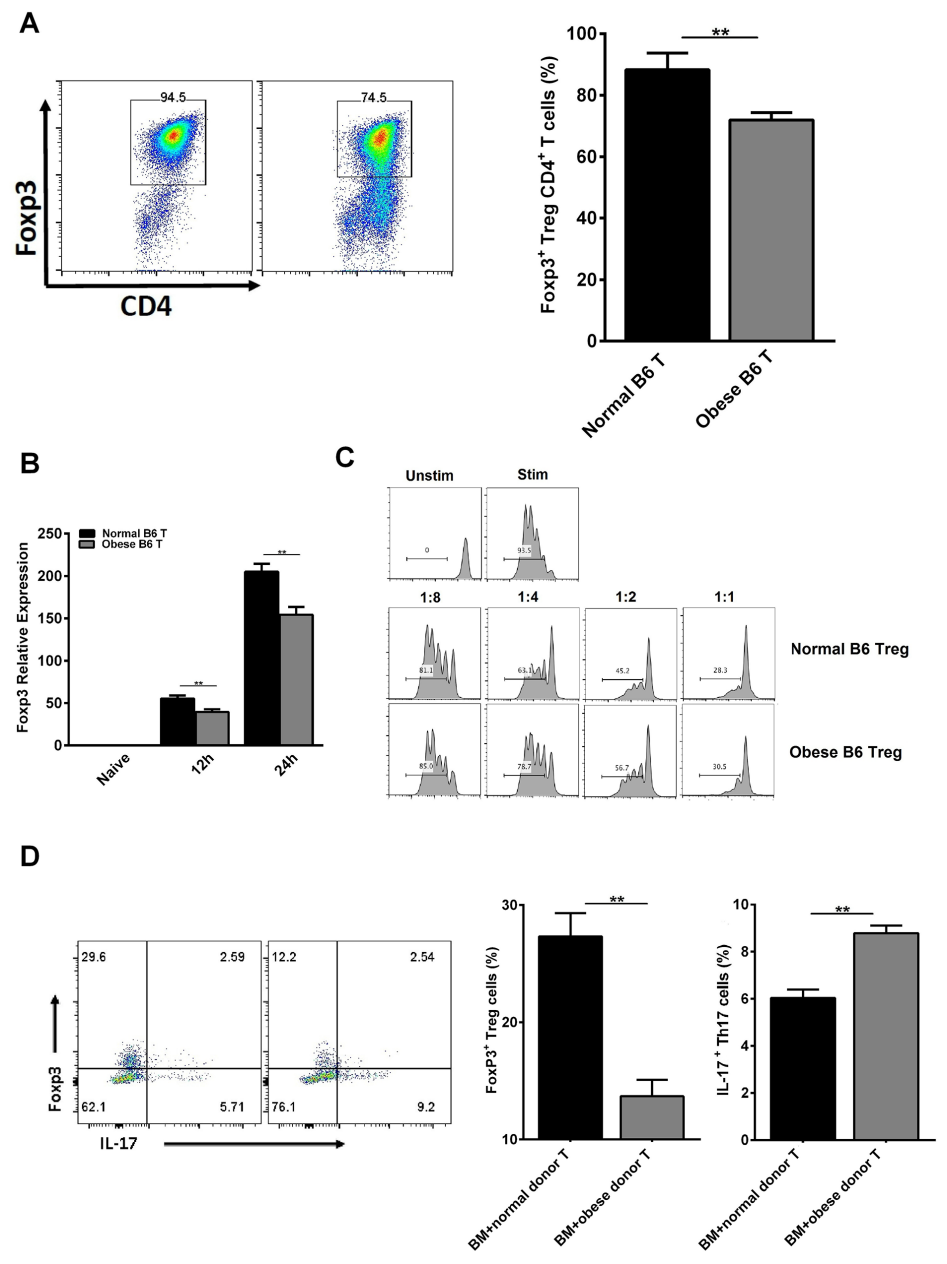
Obese donor leads to increased Th17 cells differentiation but inhibited Tregs differentiation and functional activity *in vitro* and *in vivo* Tregs were induced as described above for 3 days *in vitro*. Flow cytometry was used and mean ± SEM of the proportion of Tregs **(A)** is shown. **(B)** Real-time PCR analysis for Foxp3 was performed using RNA isolated from cells collected at 12h and 24h during CD4^+^ T cells’ induction. Mean ± SEM of gene expression level is represented. **(C)** For suppressive ability test, CD4^+^CD25^+^ Tregs obtained above were co-cultured with CFSE-labeled CD4^+^CD25^−^ T cells (1:1, 1:2, 1:4 and 1:8 ratio) in the presence of anti-CD3 mAb (145-2C11; Bio Express) coated at the dose of 0.5 μg/ml in 96-well flat-bottom plates. The proliferation (CFSE dilution) of CD4^+^CD25^−^ T cells was analyzed by flow cytometry. Lethally irradiated BALB/c recipients were transplanted with BM-TCD, with or without splenocytes from normal C57BL/6 and obese C57BL/6 mice respectively. Splenocytes for each group from recipient mice were harvested and analyzed by flow cytometry at day 14 after allo-HCT. Tregs from recipient mice were harvested as described above. Flow cytometry was used and mean ± SEM of the proportions of Tregs and Th17 cells from recipient mice **(D)** in spleen are shown. Data are combined from three independent experiments, ^*^P<0.05, ^**^P<0.01.

As is known, Tregs play a critical role on the maintenance of immunological homeostasis and self-tolerance in humans and mice [30]. To analyze the underlying mechanism of obese donor splenocytes in regulating severity of aGVHD, we studied the differentiation of Tregs and Th17 cells. We found that inducible Tregs population tended to decrease from obese B6 CD4^+^ T cells, compared with normal B6 CD4^+^ T cells *in vitro* (Figure 6A). Moreover, the expression of Foxp3 at 12h and 24h during CD4^+^ T cells’ induction is also investigated (Figure 6B). Compared to normal group, we found that Foxp3 expression was greatly reduced at each time point in obese donor CD4^+^ T cells’ induction group. Next, we tested the suppressive ability of CD4^+^CD25^+^ Tregs from normal and obese donor mice. The data showed that under normal culture conditions, obese donor spleen-derived CD4^+^CD25^+^ Tregs displayed a reduced ability to inhibit CD4^+^CD25^−^ T cell proliferation compared with normal donor spleen-derived CD4^+^CD25^+^ Tregs (Figure 6C). Then, we examined the proportion of Tregs and Th17 cells in the spleen of recipient mice by flow cytometry at day 14 after allo-HCT. As expected, while the proportion of Tregs in spleen was significantly decreased, the proportion of Th17 cells in spleen were greatly increased in obese donor aGVHD group, compared with normal donor aGVHD group (Figure 6D). These data suggested that Th17 cells might participate in inducing pathogenic effect in aGVHD and obesity inhibited CD4^+^CD25^+^ Tregs differentiation as well as their functional activity.

### Obese donor splenocytes enhance aGVHD severity through Akt/mTOR, S1PR1/ mTOR and smad3 signaling

**Figure 7:**
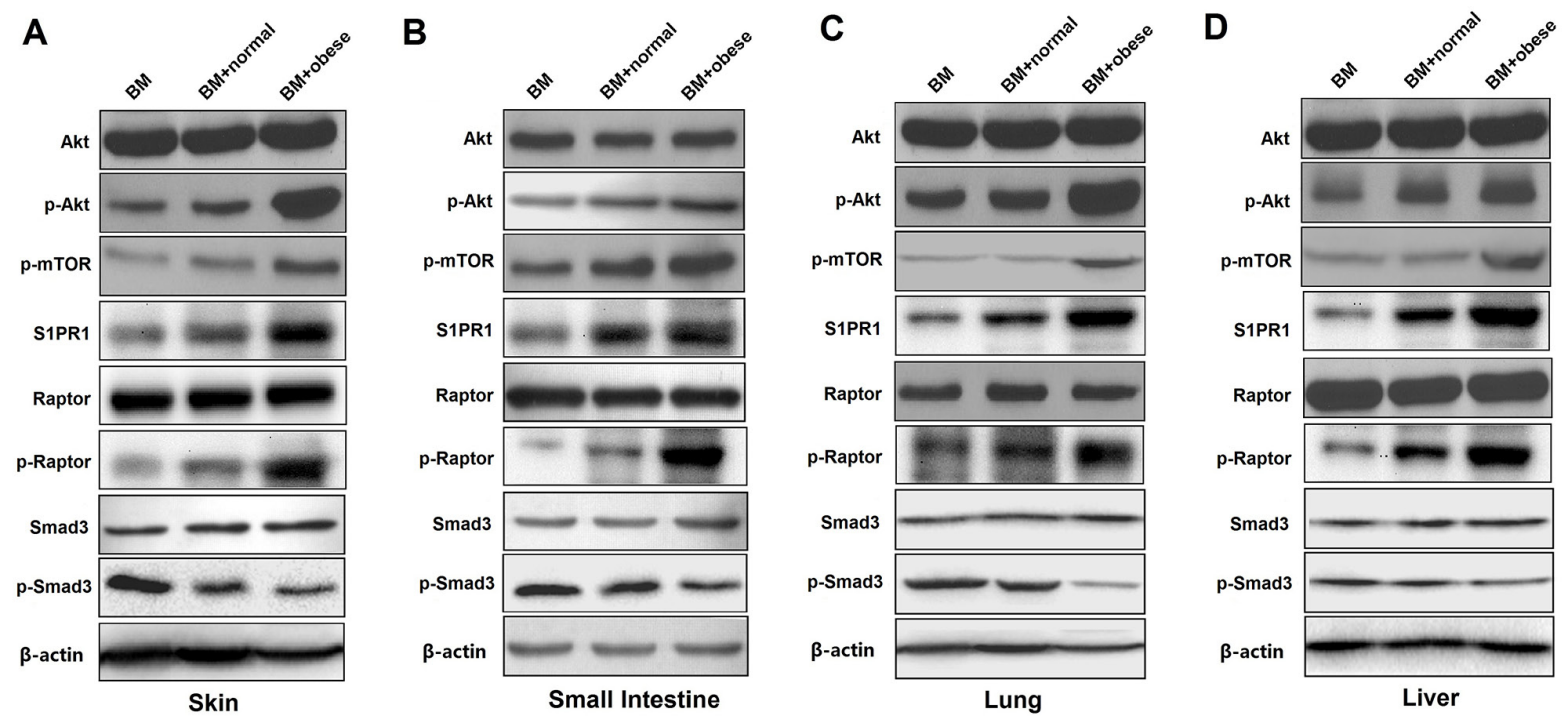
Obese donor splenocytes enhance aGVHD severity through Akt/mTOR, S1PR1/ mTOR and Smad3 signaling in the skin, small intestine, lung and liver Lethally irradiated BALB/c recipients were transplanted with BM-TCD, with or without splenocytes from normal C57BL/6 and obese C57BL/6 mice respectively. Skin, small intestine, lung and liver tissues from recipient mice were harvested at day 14 after allo-HCT. Representative examples are shown for the western blot analysis of Akt/mTOR, S1PR1/mTOR and Smad3 phosphorylation levels in total cell lysates of skin **(A)**, small intestine **(B)**, lung **(C)** and liver **(D)** tissues. All results represent at least three independent experiments.

As is known, the Akt/mTOR pathways have been reported to play prominent roles in T cell proliferation, T cell activation, migration, cytokine production and pathogenicity in the induction of aGVHD [31, 32]. S1PR1 has been shown to be important in inflammatory responses in immune cells, the decreased expression of S1PR1 can attenuate aGVHD in mouse model [33, 34]. Moreover, high level of Smad3 may correlate with low rates of both acute and chronic GVHD [35]. Therefore, we explored the roles of Akt/mTOR, S1PR1/mTOR and Smad3 signaling in the development of aGVHD in recipient skin, intestine, lung and liver tissues two weeks after allo-HCT. We examined the expression of S1PR1 and phosphorylation levels of Akt, mTOR, Raptor and Smad3 in total cell lysates of skin, intestine, lung and liver tissues by Western blot analysis (Figure 7A–7D). As expected, the expression of S1PR1 and phosphorylation levels of Akt, mTOR and Raptor were increased significantly, while the phosphorylation level of Smad3 was decreased in the skin, the small intestine, the lung and the liver from obese donor group as compared to the normal donor group. These results showed that Akt/mTOR, S1PR1/mTOR and Smad3 pathways were associated with obese donor splenocytes induced aGVHD.

## DISCUSSION

Obesity, characterized by the excess accumulation of intra-abdominal body fat, is a state of chronic, low-grade pro-inflammation that derives from the excess infiltration and accumulation of neutrophils and resident macrophages within the adipose tissue [36–38]. Obesity has more than doubled in the last decades in the general population and continues to be a global health concern [14]. Currently, several studies have demonstrated that immunological responses of obese individuals differ from that of lean individuals, and obesity increases the risk of autoimmune diseases [39–41]. The balance between pro-inflammatory and anti-inflammatory T cells is modified during obesity-induced inflammation, the pool of pro-inflammatory T cells such as CD4^+^ and CD8^+^ T cells is increased and anti-inflammatory T cells such as Tregs are decreased in diet-induced obesity [42–44]. Recent study also has shown that hyperglycemia of recipients is associated with an increased risk of aGVHD and subsequent nonrelapse mortality [45]. Therefore, these studies indicate that obesity obviously is associated with an increased risk of hyperglycemia, which can result in elevated levels of several cytokines and lead to an inferior outcome after allo-HSCT [46, 47]. In addition, it has become clear that adipocytokines, which are secreted mainly from adipocytes, play important roles in the control of immunity [48]. Particularly, the level of leptin has been found to be proportional to body fat weight and to affect Tregs proliferation as well as its function [49, 50]. Hence, it could be hypothesized that in obese recipients, a higher leptin level suppresses Treg activity, increasing the risk of aGVHD. Obesity for recipients is considered a risk factor for aGVHD, however, since obesity is a global epidemic, whether obesity for donor can affect the development of aGVHD is still unclear. In this study, we evaluated the *in vivo* and *in vitro* effects of obese donor splenocytes on aGVHD. *In vitro*, obese donor mouse CD4^+^ T cell promoted the production of IL-2, IFN-γ and TNF-α, and decreased the inducible Tregs population greatly. Then we designed a mouse aGVHD model with BALB/c mice as recipients, normal and obese B6 mice as donors, and found that obese donor splenocytes aggravated aGVHD severity and the associated weight loss. We also pointed out that there were significantly lower long-term survival rates and higher GVHD clinical pathological scores in obese donor group. Based on our results, we proposed several possible mechanisms by which obese donor splenocytes increased aGVHD severity, including increase of pro-inflammatory cytokine and chemokine’s production, promotion of CD3^+^ T cells’ expansion, regulation of Tregs subsets differentiation, enhancement of phosphorylation level of AKT and mTOR. To our knowledge, this is the first report that provides evidence suggesting that obese donor splenocytes could aggravate the pathogenesis of aGVHD through regulating CD4^+^ T cell induced-type I inflammation and differentiation of Tregs.

As is known, cytokines and chemokines are the major players in aGVHD, which are associated with target-cell tissue damage [51]. Release of these inflammatory cytokines, including IFN-γ, TNF-α, IL-2 and IL-17 into the serum during conditioning has been correlated with an increased risk of more severe GVHD and poorer survival [52–55]. Chemokine expression can be enhanced by inflammatory cytokines, and chemokines have an important role in recruiting cells of the innate and adaptive immune system to the sites of inflammation [56–59]. Previous studies have demonstrated that expression of the IFN-γ-inducible chemokines, CXCL9, CXCL10, CXCL11, CCL3 and chemokines receptor CXCR3 in aGVHD target organs has been correlated with aGVHD severity [10]. A number of studies have also shown that high expression level of CXCL9, CXCL10, CXCL11 and CCL3 plays a key role in the development of aGVHD [58, 60, 61]. Recent evidence indicates that the balance between Th1/Th2 inflammatory is of critical importance for the outcome of obesity-related metabolic disorders [62]. In addition, obesity is characterized by higher TNF-α and IFN-γ levels in plasma and in adipose tissue, and there is a reduction of these concentrations with weight loss [63, 64]. Our data indicate that obese donor splenocytes produce higher level of the pro-inflammatory cytokine IL-2, IL-17, IFN-γ and TNF-α *in vitro* and *in vivo*, followed by promoting tissue damage of skin, gut, lung and liver, compared with normal donor splenocytes (Figures 2C, 3D, 4A). Obesity for donor is also related to CD4^+^ T cell–mediated aGVHD of recipients by controlling the differentiation of CD4^+^IL-2^+^, CD4^+^IFN-γ^+^ and CD4^+^TNF-α^+^ and Th17 cells (Figure 5D-5F, 6D). What’s more, we also analyzed chemokine expression patterns in aGVHD serum and target organs using the RayBio mouse chemokine array, ELISA and real-time PCR (Figure 4B-4D). As expected, the expressions of CXCR3, CXCL9, CXCL10, CXCL11 and CCL3 were significantly improved in obese donor group. Hence, owing to the direct and indirect cytotoxicity of IL-2, IL-17, IFN-γ, TNF-α, CXCR3, CXCL9, CXCL10, CXCL11 and CCL3 to the target organs, obese donor splenocytes induces more severe aGVHD, compared with normal splenocytes (Figure 3A-3H).

As a unique T-cell lineage, recent scientific investigations have discovered the possible role of Treg cell in the development of GVHD [65]. For example, Tregs are committed to suppressive functions and play a critical role in maintaining self-tolerance and immune homeostasis [66]. Donor Tregs can reliably suppress GVHD in mice [67]. Interestingly, in an obesogenic environment, it most likely would result in disrupted T cell metabolism through increased glycolytic and oxidative flux and cause impaired T cell response by promoting inflammation and reducing anti-inflammatory immune surveillance [68]. Moreover, obesity can impair the immune response, particularly inhibited differentiation and function of Treg cells [20].

In addition, several signaling pathways play a significant role in the differentiation and function of Treg cells. Firstly, active Akt/mTOR signaling suppresses the function of Tregs and significantly impairs expression of Foxp3 [69, 70]. Inhibiting AKT can attenuate aGVHD and increase Tregs [31]. Our and other group also found that active Akt signaling can aggravate the pathogenesis of aGVHD and reduce the proportion of Tregs [6, 71]. As we all know, mTOR is an evolutionarily conserved serine/threonine kinase and a critical regulator of cellular growth, proliferation and differentiation [72]. Two distinct multi-protein mTOR complexes, termed mTORC1 and mTORC2, are found in mammalian cells. Raptor, the obligate adaptor proteins, is unique components of mTORC1 [73]. As the mTORC1 inhibitor, rapamycin was approved by the FDA to prevent allograft rejection under the generic name sirolimus [74]. mTORC1 is a downstream of the AKT signaling pathway and previous studies have proposed a link between rapamycin and Treg cells. Although a recent study has demonstrated that mTORC1 is a positive regulator of Treg functions *in vivo* [75], several researches found that mTORC1 expression inhibition can attenuate the pathogenesis of aGVHD [6]. Moreover, our and other group have also shown that rapamycin can significantly enhance the percentages of Treg cells and be used to treat T-cell–mediated diseases involving aGVHD [13, 76–79]. These results suggested that mechanistic links between mTORC1 pathways and Treg cells may be different under certain physiologic or pathologic conditions. Thirdly, the S1PR1-mTORC1 signaling axis also regulates Treg differentiation and functional fates. S1PR1 signaling to mTORC1 restrains Tregs differentiation and limits their suppressive functions *in vitro* and *in vivo* during homeostasis and inflammation [80, 81]. S1PR1 has been shown to be important in inflammatory responses and the decreased expression of S1PR1 can attenuates murine aGVHD [33, 34]. Finally, Smad3, a key transcription factor, mediates TGF-β signaling and regulates Foxp3 expression in iTreg differentiation. The activated Akt in turn interacts with Smad3, resulting in the inhibition of Smad3 phosphorylation and consequently the reduction of p-Smad3, which results in the decreased binding to the specific binding site of the foxp3 promoter [82]. Some data support the concept that Smad3 inhibition would increase Tregs and be attractive for prevention of GVHD [83]. In our study, we observed that obese donor is related to the pathogenesis of aGVHD by regulating differentiation of Tregs. *In vitro*, inducible Tregs population tended to decrease from obese donor CD4^+^ T cells, compared with normal donor CD4^+^ T cells (Figure 6A). Moreover, the expression of Foxp3 at 12h and 24h during CD4^+^ T cells’ induction is also investigated (Figure 6B). Compared to normal group, we found that Foxp3 expression was greatly reduced at each time point in obese donor CD4^+^ T cells’ induction group. Next, we tested the suppressive ability of CD4^+^CD25^+^ Tregs from normal and obese donor mice and found that obese donor spleen-derived CD4^+^CD25^+^ Tregs displayed a reduced ability to inhibit CD4^+^CD25^−^ T cell proliferation compared with normal donor spleen-derived CD4^+^CD25^+^ Tregs (Figure 6C). *In vivo*, the proportion of Tregs in spleen was significantly decreased in obese donor aGVHD group, compared with normal donor aGVHD group (Figure 6D). We also observed that the expression of S1PR1 and phosphorylation levels of Akt, mTOR and Raptor were increased significantly, while the phosphorylation level of Smad3 was decreased in the skin, the small intestine, the lung and the liver from obese donor group as compared to the normal donor group (Figure 7A-7D). Hence, these findings suggest that decreased Smad3 and increased Akt/mTORC1 and S1PR1/mTORC1 signaling result in down-regulating immune tolerance and the enhanced severity of aGVHD in animal models and explain why obese donor splenocytes produce a stronger pathogenic effect in aGVHD.

In summary, our data showed that obese donor aggravated acute GVHD severity through regulating CD4^+^ T cell induced-type I inflammation and differentiation of Tregs. Further investigation will be required to clarify the precise effect of obese donor splenocytes induced aGVHD and the underlying mechanisms.

## MATERIALS AND METHODS

### Mice

C57BL/6 (B6; H-2Kb), 6weeks old, and BALB/c (H-2Kd) mice, 8–12 weeks old, were purchased from animal core facility of Nanjing Medical University (Nanjing, China). Obese mice model using high fat diet (HFD) to induce obesity protocols as previously described [84]. Briefly, B6 mice were divided into two groups, one group fed a normal diet, and another group fed HFD (research diet, USA). All of C57BL/6 mice were fed for 12 weeks. Body weight was measured every week. BALB/c mice were fed with mouse chow and tap water ad libitum. All of the mice were maintained under pathogen-free conditions in an animal facility with controlled humidity (55±5%), light (12h/12h light/dark), and temperature (23±1°C). The air in the facility was passed through a HEPA filter system designed to exclude bacteria and viruses. The protocols used in this study were approved by the Institutional Animal Care and Use Committees at Nanjing Medical University.

### Metabolic parameters

Body weights were measured once every 4 weeks. Mice were fasted for 4h, and glucose levels (mmol/L) were determined using the Accu-Chek Performa glucometer (Roche Diagnostics, Mannheim, Germany). Serum concentrations of triglycerides and total cholesterol were measured using the Olympus AU600 Chemistry Immuno Analyzer (Olympus, Tokyo, Japan).

### BMT model and histopathology scoring

MHC and minor histocompatibility Ag (miHA)–mismatched (C57BL/6→BALB/c) bone marrow transplantation (BMT) models were used as previously established [55]. Briefly, Recipients (BALB/c) mice were conditioned with total body irradiation at 750 cGy using an RS 2000 Pro x-ray irradiator (Rad Source Technologies, USA) at the Nanjing Medical University. After conditioning, recipients were injected intraveneously (i.v.) with 5×10^6^ total T cell–depleted (TCD) bone marrow cells (C57BL/6 mice) alone with or without 1×10^6^ splenocytes from normal C57BL/6 or obese C57BL/6 mice. All experiments were performed at least three times with ten mice per group. Survival after BMT was monitored every day, and the degree of clinical aGVHD was assessed using a scoring system that summed changes in five clinical parameters: weight loss, posture, activity, fur texture, and skin integrity. Individual mice were scored 0–2 for each criterion and 0–10 overall [85]. Representative samples of aGVHD target organs (skin, intestine, lung and liver) were excised from recipients 14 d after BMT and subjected to histopathological scoring as previously described [86].

### MLR culture *in vitro*

Splenocytes derived from BALB/c mice were used as stimulator cells and those from normal C57BL/6 or obese C57BL/6 mice were used as responder cells in mixed lymphocyte reaction (MLR) assays. Splenocytes were harvested in ACK lysis buffer (eBioscience, USA), washed, and resuspended in complete culture medium (RPMI 1640 medium supplemented with 10% heat-inactivated fetal calf serum, 1 mM sodium pyruvate, 5×10^5^ M 2-ME and 20 mM HEPES). Aliquots of 2×10^5^ CD4^+^ T cells (responders) were cultured with 2×10^5^ irradiated (2500 cGy) antigen-presenting cells (APC) in 96-well plates containing 200 of complete medium at 37°C in a humidified 5% CO_2_ atmosphere. After 3 days of culture, cell culture supernatant was collected.

### Flow cytometry and cytokine detection

Single-cell suspensions of the spleen was prepared passing through sterile mesh filters and stained with various combinations of fluorescent antibodies against CD3, CD4, CD25, IL-2, IFN-γ, TNF-α, IL-17 and Foxp3 using standard flow cytometric protocols. Briefly, T cells were isolated from spleens at day 14 after BMT, then stimulated with PMA (50 ng/ml) and ionomycin (500 ng/ml) for 5 h, brefeldin A (10 μg/ml; all from Calbiochem) for 4 h. Intracellular staining was conducted using a kit (eBioscience, USA) following the manufacturer’s protocol. Flow cytometry analysis was performed on a FACSCalibur flow cytometer (BD, Biosciences). Cytokine levels in cell culture supernatants and recipient serum were quantified using a sandwich ELISA (R&D Systems, Minneapolis, MN) according to the manufacturer’s instructions.

### Chemokine array and ELISA analysis

The profiles of chemokines from serum were measured by a Mouse Chemokine Array (RayBiotech, USA) according to the manufacturer’s instructions. The chemokines with significant differences in expression were screened out. CXCL10 levels in recipient serum were quantified using a sandwich ELISA (R&D Systems, Minneapolis, MN) according to the manufacturer’s instructions.

### T cell proliferation assays *in vitro*

CFSE (Invitrogen) labeled CD3^+^ T cells from normal C57BL/6 or obese C57BL/6 mice were cultured with irradiated non-T cells (2600cGy) (1:1), while anti-CD3 mAb (145-2C11; Bio Express) were added at the dose of 0.5 μg/ml. After 3 days of culture, the proliferation of effective T cells was evaluated by CFSE dilution by flow cytometry.

### Murine T cell isolation and differentiation

Naive CD4^+^ T cells from splenocytes were purified by positive selection using CD4^+^ Naive T cells kit (Miltenyi Biotec). To obtain iTregs, isolated Naive CD4^+^ T cells from normal C57BL/6 or obese C57BL/6 were cultured in the presence of plate-bound anti-CD3 (1μg/mL; BD PharMingen) and anti-CD28 (1μg/mL; BD PharMingen), transforming growth factor β (TGF-β; 5ng/mL; PeproTech) and IL-2 (100U/ml) for 4 days. The induced Tregs were analyzed by flow cytometry.

### Treg cell suppression assay *in vitro*

CD4^+^CD25^+^ Tregs were isolated from the spleen of normal C57BL/6 or obese C57BL/6 mice through magnetic selection with CD4^+^CD25^+^ T cell isolation kit (Miltenyi Biotec) and washed with culture medium. Then Tregs were added to CFSE (Invitrogen)-labeled CD4^+^CD25^−^T cells (2×10^5^) (1:1, 1:2, 1:4 and 1:8 ratio) from C57BL/6 mice that were activated by anti-CD3 mAb (145-2C11; Bio Express) coated at the dose of 0.5 μg/ml in 96-well flat-bottom plates. The ability of Tregs to inhibit the activation and proliferation of CD4^+^CD25^−^ T cells was determined by measuring the proliferation of CFSE-labeled CD4^+^ T cells via flow cytometry.

### Real-time PCR

Before RNA extraction, tissue samples were homogenized using FastPrep-24 system (MP Biomedicals Europe, Illkirch, France). Total RNA was isolated using TRIzol reagent (Invitrogen, Carlsbad, CA) prior to complementary DNA synthesis. PCR amplification was carried out using specific primer pairs. GAPDH was used as an internal standard. The primer sequences used were obtained from Primer Bank (http://pga.mgh.harvard.edu/primerbank) and were as follows:

IL-2: 5′-GAGCAGGATGGAGAATTACAGG-3′ (sense) and 5′-CGCAGAGGTCCAAGTTCATC-3′ (antisense);

IL-17: 5′-CCTCAAAGCTCAGCGTGTCC-3′ (sense) and 5′-GAGCTCACTTTTGCGCCAAG-3′ (antisense);

IFN-γ: 5′-GGTCAACAACCCACAGGTC-3′ (sense) and 5′-GACTCCTTTTCCGCTTCCT-3′ (antisense);

TNF-α: 5′-AGGCACTCCCCCAAAAGA-3′ (sense) and 5′-CAGTAGACAGAAGAGCGTGGTG-3′ (antisense);

CXCL9: 5′-TGGGCATCATCTTCCTGGAG-3′ (sense) and 5′-CCGGATCTAGGCAGGTTTG A-3′ (antisense);

CXCL11: 5′-CGGGATGAAAGCCGTCAA-3′ (sense) and 5′-TATGAGGCGAGCTTGCTTGG-3′ (antisense);

CXCL13: 5′-AGGCTCAGCACAGCAACG-3′ (sense) and 5′-TCTTTGTAACCATTTGGCACG-3′ (antisense);

CCL3: 5′-TGCCTGCTGCTTCTCCTACA-3′ (sense) and 5′-TGGACCCAGGTCTCTTTGGA-3′ (antisense);

CXCL10: 5′-CCTCATCCTGCTGGGTCTG-3′ (sense) and 5′-CTCAACACGTGGGCAGGA-3′ (antisense);

CXCR3: 5′-TAGTGGTGGTGGCAGCCTTT-3′ (sense) and 5′-AGGCATAGAGCAGCGGATTG-3′ (antisense);

Foxp3: 5′-GGCCCTTCTCCAGGACAGA-3′ (sense) and 5′-GCTGATCATGGCTGGGTTGT-3′ (antisense);

GAPDH: 5′-AGTGTTTCCTCGTCCCGTAG-3′ (sense) and 5′-TGATGGCAACAATCTCCACT-3′ (antisense).

Fold changes were calculated using the comparative ΔΔCt method.

### Western blot analysis

Extraction of proteins from aGVHD tissue samples for immunoblotting was performed as previously described using a modified RIPA buffer [87]. Briefly, protein was extracted from tissues after homogenized by FastPrep-24 system (MP Biomedicals Europe, Illkirch, France). Total protein was isolated using ice-cold RIPA buffer (50 mM Tris, pH 7.2, 150 mM NaCl, 1 % NP-40, 1 % sodium deoxycholate, 0.05 % SDS, and 1 mM PMSF) on ice. The supernatants obtained after centrifugation were quantified with an enhanced BCA protein assay kit (Beyotime, Nantong, China). Proteins were separated by sodium dodecyl sulfate poly-acrylamide gel electrophoresis (SDS-PAGE) through 10 % gel and transferred to polyvinylidene fluoride (PVDF) membrane. Membrane was blocked with 5% skim milk in PBS containing 0.1 % Tween-20 (PBST) for 1 h at room temperature. Membrane was probed with antibodies against Akt, p-Akt, mTOR, p-mTOR and β-actin overnight at 4°C. Membrane was washed three times in PBST and incubated with peroxidase-conjugated mouse or rabbit secondary antibody for 2 h at room temperature. Membrane was washed in PBST, and the detection was done using enhanced chemiluminescence. The analysis of relative protein expression level was performed using ImageJ software (MD, USA). Antibodies against S1PR1, Akt, p-Akt, mTOR, p-mTOR, Raptor, p-Raptor, Smad3, p-Smad3 and β-actin were purchased from Cell Signaling Inc (Beverly, MA).

### Statistical analysis

Data are represented as means ± SEM. Student’s t test was used for statistical analysis. Survival curves were compared using the log-rank test. A value of *P>0.05* was considered statistically significant in all the experiments.
